# Effect of Synergistic Aging on Bauxite Residue Dust Reduction Performance via the Application of Colloids, an Orthogonal Design-Based Study

**DOI:** 10.3390/polym13121986

**Published:** 2021-06-17

**Authors:** Xuhan Ding, Guang Xu, Dengfei Wang, Zhenmin Luo, Tao Wang

**Affiliations:** 1 College of Safety Science and Engineering, Xi’an University of Science and Technology, Xi’an 710054, China; dingxuhan1990@xust.edu.cn (X.D.); 19220214064@stu.xust.edu.cn (D.W.); Christfer@xust.edu.cn (T.W.); 2 Department of Mining Engineering, Missouri University of Science and Technology, Rolla, MO 65409, USA

**Keywords:** predictor, dust control performance, synergistic aging, orthogonal design, dynamic viscosity, penetration resistance

## Abstract

The application of polymer colloids is a promising approach for bauxite residue dust pollution control. However, due to the existence of synergistic aging, the efficiency of colloid dynamic viscosity to predict the dust control performance of bauxite residue is unclear. Previous studies were also rarely performed under synergistic aging conditions. Thus, this paper investigates the relationship between colloids’ viscosity and dust control performance under synergistic aging modes. Results illustrated that the binary colloid achieved better dust control performance than unitary colloid for their higher viscosity and penetration resistance. For both unitary and binary colloid, higher viscosity results in better crust strength. A logarithmic relationship was found for viscosity and dust erosion resistance under unitary aging. However, Only the dynamic viscosity of colloids in solid-liquid two-phase conditions, rather than dissolved in deionized water, can effectively predict the dust control performance under synergistic aging conditions.

## 1. Introduction

**Figure 1:**
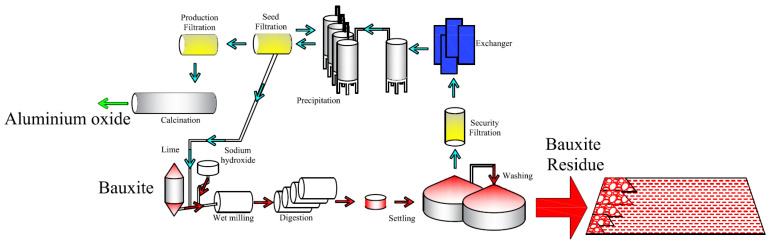
Generation of bauxite residue via Bayer process.

The bauxite production in China increased dramatically in the last several decades due to improved social demand of aluminum [1], and resulted in drastic generation of bauxite residue [2], as shown in Figure 1. By the end of 2019, Chinese annual production and gross inventory of bauxite residue have already reached to 60 Mt and 400 Mt, respectively, which accounted for over 20% of global output. Bauxite residue consists of 60% red sand fraction (>150 μm) and 40% red mud fraction (<150 μm) [3]. As bauxite residue is highly alkaline, saline and contains a certain amount of heavy metal particles and radioactive components [4], the direct discharge of bauxite residue is strongly prohibited for its detrimental occupational and environmental impacts [5,6,7], such as asthma, rhinitis, eye irritation, water pH alteration [8], soil salinity and genotoxicity [9,10]. Thus, “Dry stacking” is the most employed method for the storage and drainage of bauxite residue [11,12]. In this method, red sand is used to construct the dike walls for the consolidation of red mud.

However, if left untreated, the weak surface of red sand will be subjected to fugitive dust emission [13,14]. Previous research already indicated the potential of polymer colloids in treating red sand dust erosion by constructing surface crusts with certain thickness and stiffness [15,16,17]. A study from Kolay et al. [18] illustrated a significant improvement on sand structural strength after applying anionic inorganic acrylic co-polymers. Ayeldeen et al. [19] and Liu et al. [20] also reported that polymer colloids can effectively improve the cohesion forces of superficial sand particles. Zandieh et al. [21] and Genis et al. [22] further indicated an approximately linear relationship between polymer concentration and the corresponding structural strength of treated sand, which is also agreed with by Chen et al. [23] and Ding et al. [24]. Devrani et al. [25] and Ding et al. [26] found that the increase of polymer concentration continuously decreased the dust erosion rate of treated sand. Ding et al. [26] further stated that the fresh polymer colloids with higher viscosity can effectively enhance the penetration resistance and dust control performance. This finding is similar with the study from Lemboye et al. [27]. However, most of these studies did not consider the effect of aging, and only few of them is unitary aging based, although synergistic aging is inevitable in actual field practice.

**Figure 2:**
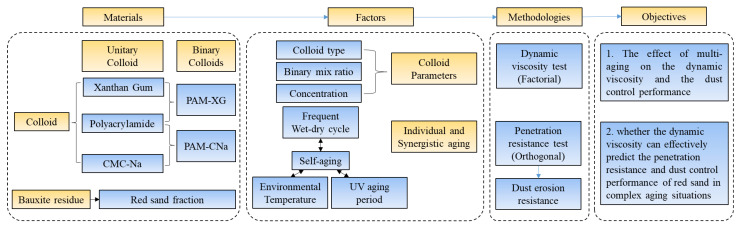
Experiment flowchart and methodology.

Thus, this paper investigates the effect of synergistic aging on the dynamic viscosity and the dust control performance of red sand when applying unitary and binary colloids, to analyze whether the dynamic viscosity can still effectively predict the penetration resistance and dust control performance of red sand. The penetration resistance of formed crust is setup as the main mechanical properties to reflect the dust control performance for their stable consistency [14,20,28]. As shown in Figure 2, both unitary colloid and binary colloids were investigated. The dynamic viscosity of colloids and the penetration resistance of red sand crust were analyzed in unitary and synergistic aging modes. Findings are helpful to study the predictor of the dust control performance under synergistic aging in the field of mining industry and environmental protection.

## 2. Materials and Methods

### 2.1. Materials

Three anionic colloids, polyacrylamide (PAM), xanthan gum (XG) and sodium carboxymethylcellulose (CNa), were utilized in this study. Among them, PAM and XG are synthetic polymer colloids, and CNa is the carboxymethylated cellulose derivatives.

PAM (C_3_H_5_NO)n is a linear synthetic polymer manufactured from acrylamide monomer via the polymerization process initiated by free radicals. It has a wide molecular weight range from 3–25 million [29]. It is applied in the fields of metal ions and organic matters removal [30,31,32], dust and soil erosion control in agriculture irrigation and arid regions [22,33,34,35] and landing pad enhancement [29] due to its thickening properties. The raw anionic 10% PAM was supplied by Aladdin Biochemical Technology Co., Ltd., Shanghai, China, in solution form.

Xanthan gum (C_35_H_49_O_29_)n is produced by the fermentation of xanthomnas campestris and carbohydrates. It is a β-D glucose with a glucuronic: D-mannose:D-glucuronic acid molar ratio of 1:1:0.7 [36]. The raw xanthan gum was supplied by Sigma Aldrich in light brown powder form.

CNa [C_6_H_7_O_2_(OH)_2_OCH_2_COONa]n is a carboxymethylated derivative of cellulose and the main anionic cellulose gum. CNa is prepared from the reaction between natural cellulose, caustic alkali and monochloroacetic acid [37,38]. In terms of its thickening, suspension, adhesion and water retention properties, it is widely applied in the fields of geology, petroleum and medicine manufacturing [39,40]. The raw CNa powder was supplied by Aladdin Pty. Ltd. All colloids were dissolved and diluted in deionized water to concentrations of 0.4, 0.8 and 1.2 wt.%.

**Figure 3:**
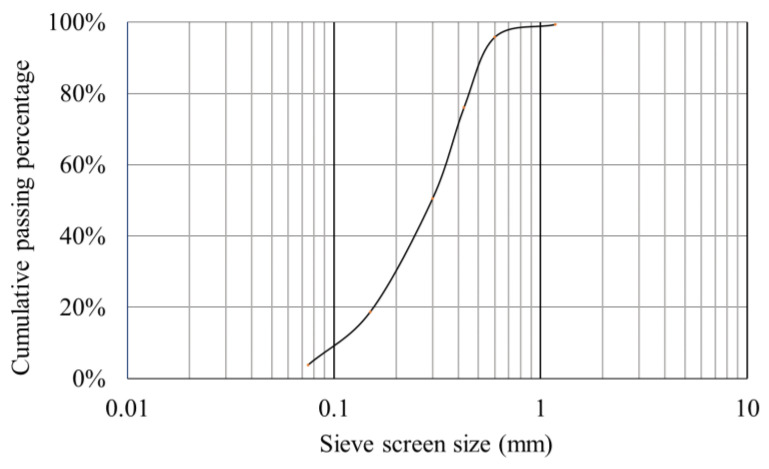
Red sand particle size distribution.

The particle size distribution of red sand is shown in Figure 3, it is defined as the poorly graded sand (SP) according to ASTM-D2487 [41]. It has an optimum moisture content (OMC) and maximum dry density (MDD) of 6.57% and 2.34 g/cm^3^, respectively, according to AS 1289.2.1.1 [42] and AS 1289.3.6.1 [43]. Its friction angle and cohesion force are 37.13° and 0.28 kPa according to AS 1289.6.2.2 [44]. The raw red sand was supported by Light Metal Research Institute, CHINALCO.

### 2.2. Dynamic Viscosity Test

The dynamic viscosity characterizes the internal friction between solvent and solute molecules by calculating the ratio of fluid shear stress to shear rate. In this study, the self-aging time were determined as 0 day and 60 days. and the effect of polymer concentrations were investigated at three levels: 0.4, 0.8 and 1.2 wt.%. For each concentration, three mix ratios were selected as 1:3, 2:2 and 3:1. Tests were performed under the ambient temperature of 20 °C to eliminate the significant effect of temperature on dynamic viscosity.

### 2.3. Aging Based Penetration Resistance Test

#### 2.3.1. Humidity Aging Test

**Figure 4:**
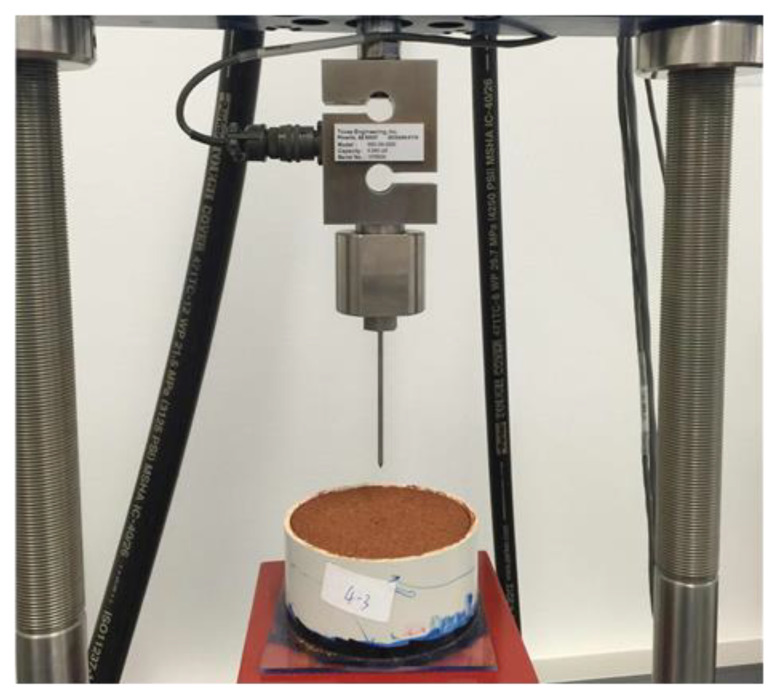
Penetration resistance test apparatus.

Humidity aging test was conducted to evaluate the water resisting capacity of PAM and its binary colloids quantified by the penetration resistance of formed crust after several humidity aging process. Four factors were considered which are binary colloid types (PAM: XG, PAM: CNa), concentrations (0.4, 0.8 and 1.2 wt.%), mix ratios (1:3, 2:2 and 3:1) and the number of cycles (0, 1 and 2). L9 (3^4^) orthogonal design was applied to obtain equivalent results with less numbers of runs. For this test, samples were prepared in 5cm (H) by 10 cm (D) cylindrical molds with a spray rate of 2 L/m^2^. After being completely dried, another 2 L/m^2^ of deionized water was sprayed on the surface of the dried crust. Samples were then sealed for 48 h and then ambient dried to accomplish one humidity aging cycle. Penetration resistance test was performed by using STX-100 hydraulic soil triaxial system and self-designed penetration connector, as shown in Figure 4.

#### 2.3.2. High Temperature Aging Test

Temperature bearing tests investigate the adaptability of binary colloids in high temperature areas. Penetration tests were conducted on samples cured for one week under 20 °C, 45 °C and 70 °C, which represent the normal, medium and high level of environmental temperatures observed onsite. Three factors were considered: the binary colloid types, colloid concentrations and mix ratios. Levels of each factor are the same with those described in Section 2.3.1. All samples were performed by following L9 (3^4^) orthogonal design. Each run was replicated 3 times.

#### 2.3.3. Ultraviolet Radiation Aging Test

This test was performed to evaluate the UV-aging resistance of the consolidated layer (formed crust) when exposed to direct solar UV radiation for months of time. The corresponding penetration resistance will quantify the service life of the binary colloids. According to ASTM G156-16 [45], UVA-340 lamp was employed to simulate the critical sunlight spectrum with short-wave wavelength range from 295 to 360 nm. 0, 7- and 14-days UV aging periods which equal to years of exposure was determined in this study. Samples were prepared and tested via L9 (3^4^) orthogonal design. Each run was replicated 3 times.

### 2.4. Orthogonal Design

The orthogonal design is one of the most important statistical experimental design methods. It can effectively evaluate the experiments with multiple factors in multiple levels. The orthogonal design can vastly reduce the number of tests via the selection of uniformly distributed factors’ combination. The result of this method is still reliable and appropriate, which can clearly represent the significance of each factor or multi-factor combination and help to determine the optimum level of each factor in terms of achieving better performance. In this study, as four factors were considered at one time and each factor has no more than 3 levels, L9 (3^4^) orthogonal design is employed.

## 3. Results and Discussions

### 3.1. Dynamic Viscosity

**Figure 5:**
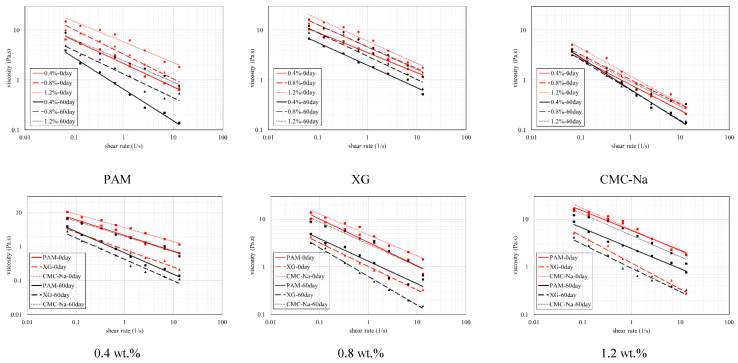
Dynamic viscosity comparison of single polymers at different aging time-nodes.

The dynamic viscosity of unitary colloid is affected by the shear rate, concentration and self-aging. As illustrated in Figure 5, the viscosity and the shear rate reveal double logarithm relationship. The viscosity decreases with higher shear rate, which exhibits pseudoplastic ability. This is mainly due to the destruction of the entanglement of polymer chains under shear stress, and the deformation of solvated layer of polymer particles, which leads to a decrease in apparent viscosity. For the same shear rate, higher concentration signifies more serious disruption of molecular network structure and more evident viscosity loss. When the shear rate increases to a certain level, the messy curly polymer molecules have been forced to stretch and reorient to the maximum extent to reach the equilibrium of apparent dynamic viscosity. After that, the decline trend of viscosity tends to constant even excessive shear rate applied.

According to the power law equation (Equation (1)) [46]:

(1) $$\eta =k{\left(\gamma \right)}^{n-1}$$

where “*k*” is the consistency coefficient and “*n*” is the flow behavior index. It is noted from Table 1 that “*k*” reduces significantly with higher concentration while no obvious change could be found for “*n*”. The same phenomenon was also observed for XG and CNa. This implies that the reduction of concentration only decreases the internal consistency of colloid. The dynamic viscosity of CNa colloid is higher than that of PAM and XG. This superiority is inconspicuous at first but tend to be more evident when higher concentrations applied. Figure 5 also denotes that self-aging reduces the dynamic viscosity significantly. CNa and XG solution reveal 20.1% to 42.8% and 29.7% to 41.5% reduction in viscosity after being aged for 60 days, respectively. The effect of self-aging is more apparent for PAM solution as its viscosity suffers a loss of 57.6% to 66.8%.

**Figure 6:**
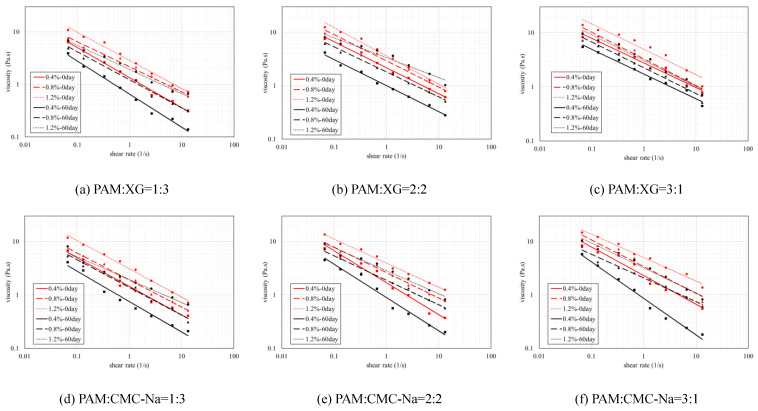
Dynamic viscosity of polymer mixtures at different aging times.

The dynamic viscosity of binary colloid is also affected by the concentration and self-aging. Figure 6 illustrated the dynamic viscosity of binary colloids at different concentrations and aging time. Results indicated that the viscosity increases with higher concentration but decreases after self-aging occurs (dotted lines and red lines are always higher). Meanwhile, the improve rate of dynamic viscosity is also affected by the aging time. As shown in Table 2, if no self-aging occurs, all binary colloids reveal slight improvement on dynamic viscosity when concentration increases from 0.4 wt.% to 0.8 wt.%, and then improve more rapidly when higher concentration applied. In contrast, binary colloids aged for 60 days demonstrate entirely different trend on viscosity enhancement. In this condition, obvious improvement on viscosity at lower concentration was observed but then tend to inconspicuous at higher concentration.

**Figure 7:**
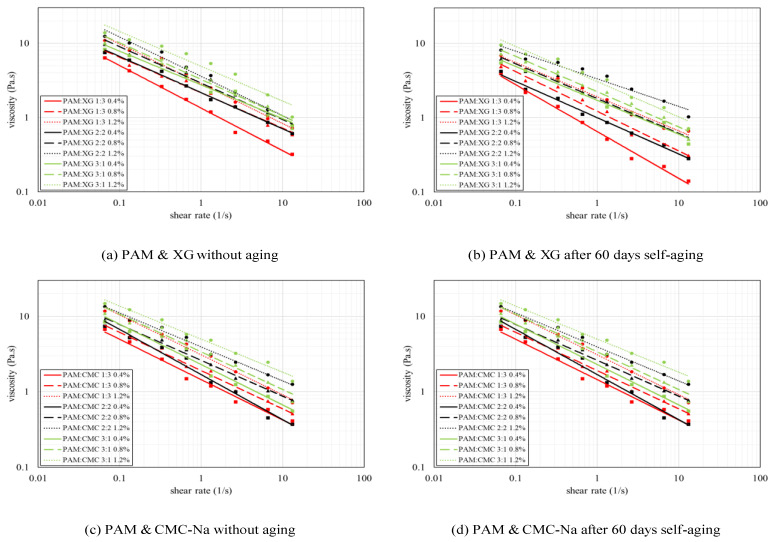
Dynamic viscosity of polymer mixtures at different mix ratios.

The viscosity of binary colloids is not necessarily higher than that of each single component. As shown in Figure 7, the viscosities of all binary colloids increase with higher PAM content (the green line with highest PAM content is higher than the other two). However, PAM-XG mixture illustrates higher viscosity compared with single PAM and XG solution, whereas PAM-CNa mixture reveals slight reduction in dynamic viscosity. This phenomenon may be explained by the existence of entanglement concentration and the critical molecular weight. In this study, the tested concentration is in the range of entanglement concentration “c_e_” which was described by Wu [47]. In this situation, the macromolecular chains of polymer colloids are fully overlapped and entangled. The chain segments are roughly and evenly distributed to form 3D entangled networks. After the concentration reaches the critical “c_e_”, further addition of polymers will reduce the dynamic viscosity of the colloids system instead of enhancing it. As “c_e_” is mostly affected by the molecular weight as higher molecular weight equals to higher “c_e_”, the concentration of PAM-XG colloid does not reach its critical “c_e_” for its relative higher molecular weight compare with PAM-CNa colloid.

### 3.2. Self-Aging vs. Unitary Colloid

The effect of self-aging is significant to the penetration resistance of red sand samples treated by unitary colloid, and this effect is more obvious at higher concentrations. Table 3 illustrated the results of self-aging test when applying unitary colloids onto red sand surface. Both the effects of each single factor (A, B and C) and two factors interaction (B*C) are analyzed. The absolute range reflects the significance of each factor which refers to the difference between the maximum and the minimum “kn” values. It is noted that the self-aging is more significant than colloid type and concentration for its relative higher absolute range values.

The effect of B*C on the penetration resistance can be clearly separated into two stages. For concentration increases from 0.4 to 0.8 wt.% (the level of Factor B from 1 to 2), B*C reveals an absolute range of 2.110 which is insignificant to the red sand structure strength. However, by further increasing the concentration from 0.8 to 1.2 wt.% concentration, this absolute range increase to 6.190 rapidly which represent a more significant effect. Thus, although the individual effect of concentration and self-aging on red sand penetration resistance is significant, but the effect of their interactions is diminished at low concentration range and tend to be enhanced at higher concentrations. On the other hand, the effect of polymer concentration on penetration resistance is strengthened with longer self-aging period, which denoted that to achieve desired red sand structure strength, higher concentration is essential for polymers that aged for a long period.

### 3.3. Self-Aging vs. Binary Colloid

Different with unitary colloids, the effect of each factor and their two-terms interactions on penetration resistance varies a lot for binary colloids. For instance, in terms of the absolute range value of 1.262, the type of binary colloids can hardly influence the penetration resistance. This is because the effect of PAM on the viscosity of binary colloid is extremely significant, which directly weakens the role of other polymers in the binary colloid systems.

The penetration resistance is becoming more sensitive to colloids concentration and self-aging in the binary colloid system. The absolute range of concentrations and self-aging are 11.507 and 8.683, respectively, which is significantly higher than other factors. It could also be noted from Table 4 that the absolute value of the mix ratio is 7.940. For the same condition, the colloid with higher PAM content represents higher penetration resistance.

**Figure 8:**
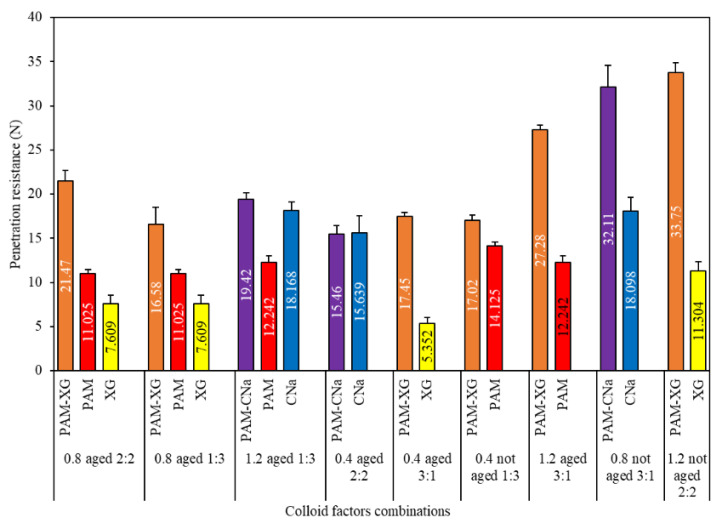
Penetration resistance due to unitary and binary colloids.

Although the penetration resistance of red sand treated by binary colloids is almost higher than that of unitary colloid, one exception was observed for 0.4 wt.% PAM-CNa colloid aged for 60 days. As shown in Figure 8, one hypothesis can be made by considering the interaction effect of “B&D” and “C&D” is that the effect of synergistic application of colloids on the penetration resistance is simultaneously constrained by the concentration, mix ratio and self-aging. The highest penetration resistance is formed by binary colloids with higher PAM contents, higher concentration, and less self-aging period. However, if the dynamic viscosity of one additive is evidently higher than that of PAM, the binary colloid system will not achieve higher penetration resistance at low PAM content after aged for a long period. In this study, the absolute range for factor A is only 1.262, which denote insignificant effect on penetration resistance. The highest penetration can be achieved at the concentration of 1.2 wt.% and the mix ratio of 3:1 with no self-aging.

### 3.4. Synergistic Aging vs. Unitary Colloid

#### 3.4.1. Humidity Aging Test

Table 5 illustrates the data analysis of the orthogonal experiment for the humidity aging test. The absolute range of each factor indicates that the penetration resistance is primarily affected by the type of colloids, self-aging time and humidity aging. The absolute range values for the former three factors are 4.088, 1.626 and 2.688, respectively, which is significantly higher than that of colloid concentration. Compares with the results presented in Section 3.2, the effect of colloid type remains at a high level, but the effect of colloid concentration is diminished from 1.780 to 0.924. This is essentially due to the degradation of formed microstructure after long term synergistic aging. This is because for each colloid, the single, linear and longer polymer chain is generated and aggregated to emerge film or filamentous structure among red sand particles when initially sprayed onto red sand surface. If no aging occurs, the hydroxyl and carboxyl groups is easy to interact with the metal cations of red sand, and the formed crust with certain thickness and strength is insoluble and irreversible. However, due to the synergistic effect of self and humidity aging, the degradation of polymer chain results in the partial failure of the microstructure of formed crust. Different with the research from Ding et al. [26], the red sand sample were sealed for 48 h in this study to allow sufficient interaction between red sand particles and degraded colloid component. As the presence of self-aging accelerated and enlarged the hydrolysis of amide groups, the penetration resistance of formed crust tends to weaker and is more sensitive to frequent wetting.

**Figure 9:**
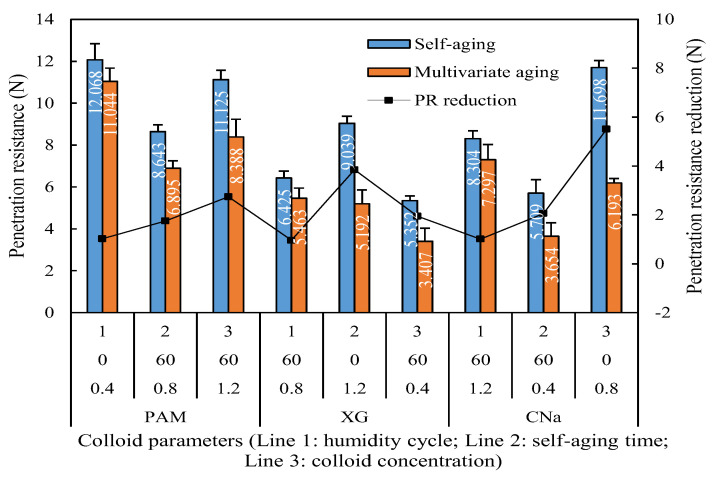
Humidity aged penetration resistance reduction for individual application.

The penetration resistance comparison between self-aging and synergistic aging is shown in Figure 9, which illustrated that the penetration resistance of red sand treated by synergistic aging is evidently lower than that of self-aging. The x-axis refers to the runs in Table 5. The penetration resistance of samples treated by synergistic aging reveals 8.49% to 42.56% reduction than those which are cured by self-aging.

#### 3.4.2. High Temperature Aging Test

The data analysis of the orthogonal experiment for temperature aging test is illustrated in Table 6. The effect of colloid type and concentration on penetration resistance remains approximately consistent when compared with the humidity aging test, but the effect of each aging factor on penetration resistance changes evidently. The absolute range value of self-aging and temperature aging in this test is 3.028 and 0.981, respectively, which is entirely different with that of humidity aging test presented in Table 5. For samples cured by high temperature, the effect of self-aging on penetration resistance is enhanced while the effect of temperature is diminished.

**Figure 10:**
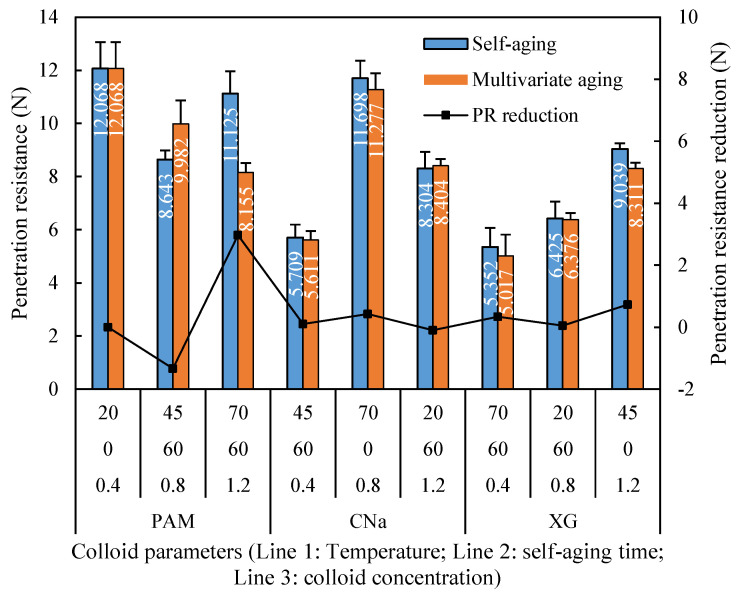
Temperature aged penetration resistance reduction for individual application.

Figure 10 provides a comparison of the crust penetration resistances between self-aging and synergistic aging. It is noted that the penetration resistance of samples treated by synergistic aging is mostly lower than that of self-aging. The x-axis refers to the runs in Table 6. It is noted that the penetration reduction for XG and CNa treated samples are 0.76% to 8.05%, respectively. However, the PAM treated sample is more sensitive to the temperature. It can be seen from Figure 10 that the penetration resistance of crust formed by PAM at 45 °C has an average penetration resistance of 9.982 N, which is 15.49% higher than that cured at ambient 20 °C temperature. However, when temperature continuously increases to 75 °C, the corresponding penetration resistance is only 8.155 N, which is 26.70% lower than that cured at 20 °C.

The effect of synergistic aging in this test stable when experimental temperature increases from 20 °C to 70 °C, which is different with the phenomenon observed in humidity aging test. This phenomenon can be explained by the existence of conformation transition temperature (CTT). CTT refers to the critical temperature to cause the disordering, instability and viscosity reduction of colloid from ordering, stability and viscosity enhancement. It was confirmed that the CTT of PAM is around 50 °C, and this value for XG and CNa is approximately 120 °C and 180 °C. In this test, red sand samples were high temperature treated immediately after the spray of colloids, the superficial PAM layer which were not infiltrated into red sand is extremely sensitive to the environmental temperature. When the temperature exceeds CTT, PAM will obviously undergo a hydrolysis reaction due to the thermal rupture of the chemical bond, and this reaction is accelerated with the further increase of temperature. As the highest temperature applied in this study is 70 °C, PAM treated sample reveals apparent reduction in penetration resistance after temperature exceed 45 °C while that of samples treated by XG and CNa remains approximately consistent.

#### 3.4.3. Ultraviolet Radiation Aging Test

**Figure 11:**
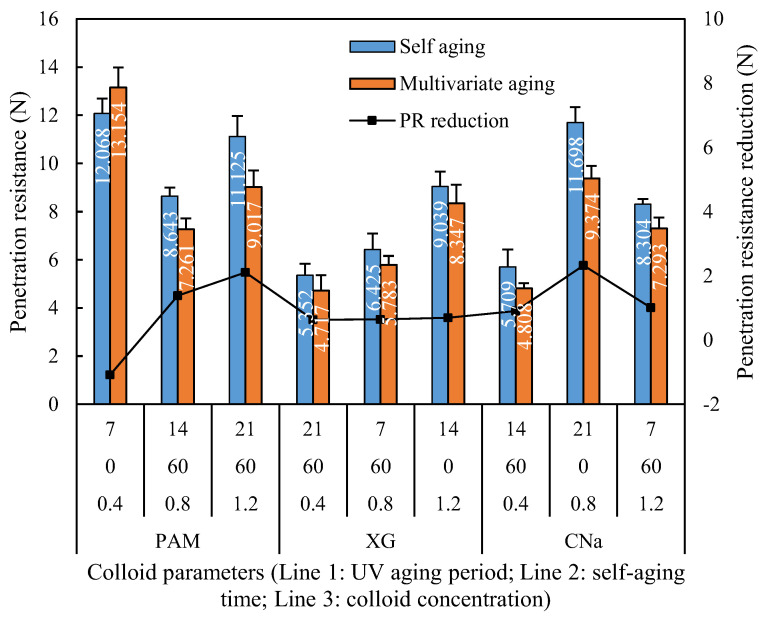
Penetration resistance reduction of UV aging test.

Figure 11 demonstrates the penetration resistance of red sand samples cured by self-aging and synergistic aging. It is apparent that the penetration resistance of PAM and XG treated samples reveals a 5.28% to 8.26% improvement after 7 days UV aging, but then starts to decrease significantly when longer UV aging applied, and finally reveals a reduction of 8.29% to 23.38%. In contrast, the penetration resistance of CNa treated sample reveals continuous reduction of 13.86% to 24.79% with longer UV aging period with no short-term penetration resistance enhancement observed.

The penetration reduction of XG treated samples was 7.66% to 11.86%, which is slightly lower than that of PAM treated samples which exhibited a 9% to 18.95% reduction. This phenomenon could be explained by the relationship between the bond energy and threshold wavelength. It is noted that the primary chemical bonds of PAM are C-C, C-N and C-H with a bond energy of 347 kJ/mol, 305 kJ/mol and 413 kJ/mol, respectively, which requires 325 nm, 369 nm and 288 nm wavelength to be broken down. However, the primary chemical bonds of XG are C-C, C-O, C=O and O-H with a bond energy of 347 kJ/mol, 358 kJ/mol, 745 kJ/mol and 467 kJ/mol, respectively, which requires 325 nm, 310 nm, 151 nm and 240 nm wavelength to be broken down. As the wavelength for UVA-340 applied in this study is ranges from 315–400 nm, it can easily break down the C-C and C-N bonds in PAM, but hardly affect the C-H bond. In contrast, for XG, the radiation can only rupture the C-C and C-O bonds while C=O and O-H bonds remains. As a result, the structure strength of red sand treated by XG is relatively stable.

The data analysis of the orthogonal experiment for UV aging test is illustrated in Table 7. Similar with the Section 3.4.1 and Section 3.4.2, the colloid type and self-aging significantly affects the corresponding penetration resistance, while the effect of colloid concentration is diminished. The absolute range of colloid type and self-aging is 3.195 and 3.645, respectively, which is obvious higher than that of colloid concentration. However, from the perspective of the interaction between self and UV aging (C&D), it is noted that the effect of synergistic aging on final penetration resistance is more significant when UV aging period increases from 7 to 14 days and then tends to inconspicuous when further increases to 21 days, which is similar with the results of humidity aging test.

### 3.5. Consistency between Viscosity and Penetration Resistance

**Figure 12:**
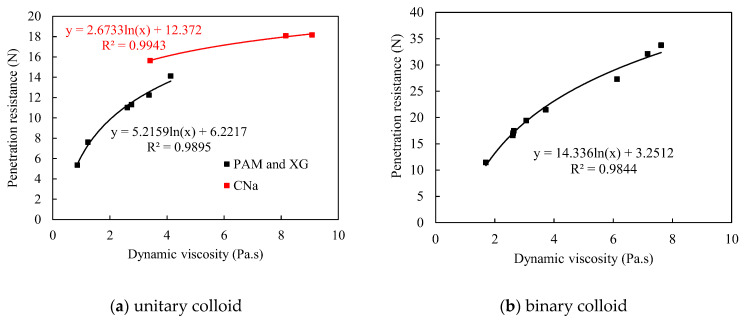
Penetration resistance vs. dynamic viscosity during multivariate aging.

The unitary colloids reveal acceptable consistency between viscosity and corresponding penetration resistance, which partially agrees with the findings from Ding et al. [26]. As shown in Figure 12a, the penetration resistance increases with higher dynamic viscosity for each unitary colloid, and the relationship between dynamic viscosity and the corresponding penetration resistance is logarithmic, which is similar with the result from Ding [26]. However, surprisingly, a sudden increase of penetration resistance for CNa treated red sand is observed despite of the reduced viscosity (red dots). As a result, the consistency of penetration resistance and viscosity is not strictly retained. One possible explanation to the reduced viscosity is due to the pH values and salinities of red sand. For low pH and salinity environment, the CNa colloid exhibits strong polyelectrolyte properties. However, by further increase the pH value and salinity, the macromolecules of CNa colloid are shielded by metal salt hydronium and thus exhibit typical non-polyelectrolyte ability, which results in the rapid increase of viscosity. However, after the pH value exceed 10, more complex chemical and physical reactions occur due to the influence of the concentration of metal ions, and, finally, causes evident decrease of dynamic viscosity.

In contrast, the binary colloids reveal excellent consistency between dynamic viscosity and penetration resistance. Figure 12b illustrated the change in penetration resistance for colloids with different viscosities at the shear rate of 0.33/s, it is noted that all binary colloids with higher viscosities reveals better crust strength, and the relationship are logarithmic, which complies the conclusion in other studies [26]. Thus, dynamic viscosity of binary colloids is sufficient to predict the penetration resistance and reflect the dust control performance accordingly when self-aging occurs individually.

**Figure 13:**
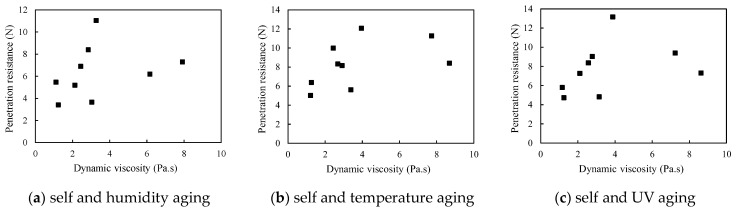
Penetration resistance vs. dynamic viscosity during multivariate aging.

The multivariate aging could result in completely inconsistency between the dynamic viscosity of binary colloid and corresponding penetration resistance. As shown in Figure 13, all the penetration resistance values are randomly distributed. It was also confirmed by the ANOVA analysis presented in Table 8 that the p(F) values for each multivariate aging test are higher than 0.05. This denoted that the dynamic viscosity of colloids is insufficient to be the key index for the prediction of dust control performance when multivariate aging occurs.

**Figure 14:**
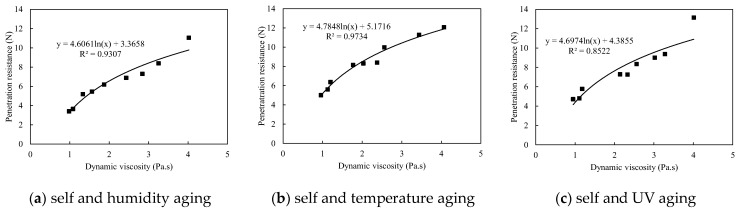
Penetration resistance vs. dynamic viscosity (red mud leaching solution based).

However, when the colloid is sprayed on the surface of the red sand, it transferred from single liquid or solid phase to solid-liquid two-phase. Due to the strong alkalinity of the red sand, the viscosity of binary colloid in solid-liquid two-phase will change greatly. In this case, using the viscosity of the raw binary colloid to predict penetration resistance and wind erosion resistance will inevitably results in significant errors. Thus, the red mud leaching solution was applied as solvent instead of solid-liquid two-phase conditions to further determine the actual dynamic viscosity of the binary colloid for the similar pH environment and reduced contents of metal ions, as shown in Table 9. The dynamic viscosity was measured by adding 100 g of colloid into 200 g leaching solution. High speed electromagnetic agitator was applied to ensure uniform dispersion. Figure 14 illustrated the relationship between the dynamic viscosity (red mud leaching solution based) and the corresponding penetration resistance. It is noted that colloid with higher viscosities depicts higher crust strength and consequently stands for better dust erosion resistance. The relationships between viscosity and penetration resistance are logarithmic. Thus, the dynamic viscosity of colloids in solid-liquid two-phase conditions rather than dissolved in deionized water, can effectively predict the dust control performance under different aging conditions in a timely and cost-effective manner.

## 4. Conclusions

This study indicated that the application of binary colloids can achieve better dust control performance. The viscosity of binary colloids increases with higher PAM content. The improve rate of colloid viscosities reveals completely opposite trend with or without self-aging. The overall viscosity reduction for PAM, XG and CNa colloids aged for 60 days are 57.6% to 66.8%, 29.7% to 41.5% and 20.1% to 42.8%, respectively.

For unitary self-aging, it can reduce the penetration resistance of treated red sand, and this effect is more obvious at higher colloid concentration. Especially for binary colloid system, as the absolute range of aging times increased from 2.837 to 8.683 rapidly, therefore the penetration resistance of samples treated by binary colloids is more sensitive to self-aging. For synergistic aging, the penetration resistance is further reduced compared with self-aging. In humidity aging test, the penetration resistance of samples treated by synergistic aging reveals 8.49% to 42.56% reduction than those which are cured by self-aging. In high temperature aging test, due to the existence of CTT, the interaction of synergistic aging on affecting the penetration resistance is approximately stable when a higher temperature is applied. In UV aging test, the penetration resistance for PAM and XG colloids treated red sand increased firstly and then decreased at longer UV aging period, while that of CNa reveals only monotonous decrease.

According to this study, the relationship between dynamic viscosity and the corresponding penetration resistance is approximately logarithmic for both unitary and binary colloids in individual aging mode. However, the dynamic viscosity of colloid when dissolved in deionized water can no longer predict the dust control performance because of physicochemical properties of red sand in solid-liquid two-phase, when spray colloids onto red sand surface. Only the dynamic viscosity of colloids in solid-liquid two-phase conditions can still be able to predict the dust control performance effectively under different aging conditions.

Findings and research method of this study offers a timely and cost effectively approach for the extended dust control study in the field of mining and environmental protection. More future work is necessary to study the effect of multivariate aging on binary colloid system in reducing red sand dust control, for the purpose of establishing more sound and systematic evaluation strategy.

## Figures and Tables

**Table 1:** Fluid parameters of polymer solution before and after self-aging.

**PAM con. (wt.%)**	**0.4**		**0.8**		**1.2**	
**Aging time (day)**	0	60	0	60	0	60
***k***	2.215	0.651	3.267	1.314	5.777	2.435
***n***	0.536	0.370	0.508	0.527	0.586	0.576
***η***	4.13	1.42	6.15	2.61	10.16	3.37
**XG con. (wt.%)**	**0.4**		**0.8**		**1.2**	
**Aging time (day)**	0	60	0	60	0	60
***k***	0.810	0.651	1.024	0.631	1.209	0.943
***n***	0.489	0.370	0.506	0.397	0.451	0.504
***η***	1.47	0.86	1.75	1.23	2.76	1.76
**CNa con. (wt.%)**	**0.4**		**0.8**		**1.2**	
**Aging time (day)**	0	60	0	60	0	60
***k***	3.555	1.991	4.683	3.017	6.053	4.584
***n***	0.598	0.549	0.562	0.534	0.553	0.551
***η***	5.96	3.41	8.16	5.62	11.43	9.08

**Table 2:** Improve rate of dynamic viscosity at different aging times.

**PAM:XG**				
**Mix Ratio**	**0 day**		**60 days**	
	**0.4 wt.%→0.8 wt.%**	**0.8 wt.%→1.2 wt.%**	**0.4 wt.%→0.8 wt.%**	**0.8 wt.%→1.2 wt.%**
1:3	37.8%	73.7%	83.8%	29.1%
2:2	22.5%	48.8%	104.4%	48.4%
3:1	19.0%	48.9%	57.2%	47.7%
**PAM:CNa**				
**Mix Ratio**	**0 day**		**60 days**	
	**0.4 wt.%→0.8 wt.%**	**0.8 wt.%→1.2 wt.%**	**0.4 wt.%→0.8 wt.%**	**0.8 wt.%→1.2 wt.%**
1:3	33.5%	46.8%	119.1%	21.4%
2:2	25.0%	46.8%	62.4%	52.2%
3:1	31.1%	25.1%	63.3%	90.0%

**Table 3:** Orthogonal data analysis of self-aging test for individual polymer application.

Runs	Factors			Interactions		PR (N)
	A	B	C	B&C	B&C	Averaged
	Polymers	Con. (wt.%)	Aging Time	B from 1 to 2	B from 2 to 3	
1	1 (XG)	3 (1.2)	1 (0)		2	11.304
2	2 (CMC)	1 (0.4)	2 (60′)	2		15.639
3	1 (XG)	1 (0.4)	2 (60)	2		5.352
4	2 (CMC)	3 (1.2)	2 (60)		1	18.168
5	3 (PAM)	3 (1.2)	2 (60′)		1	12.242
6	1 (XG)	2 (0.8)	2 (60′)	1	2	7.609
7	3 (PAM)	1 (0.4)	1 (0)	1		14.125
8	3 (PAM)	2 (0.8)	2 (60)	1	2	11.025
9	2 (CMC)	2 (0.8)	1 (0)	2	1	18.098
K_1_	24.265	35.116	43.527	32.759	48.508	
K_2_	51.905	36.732	70.035	39.089	29.938	
K_3_	37.392	41.714				
k_1_	8.088	11.705	14.509	10.920	16.169	
k_2_	17.302	12.244	11.673	13.030	9.979	
k_3_	12.464	13.905	0.000	0.000	0.000	
Abs. Range	9.213	2.199	2.837	2.110	6.190	

**Table 4:** Orthogonal data analysis of self-aging test for individual polymer application.

Runs	Factors				Interactions				PR (N)
	A	B	C	D	B&D	B&D	C&D	C&D	Averaged
	Polymers	Con. (wt.%)	Mix Ratio	Aging Time	B from 1 to 2	B from 2 to 3	C from 1 to 2	C from 2 to 3	
1	2 (PAM-CMC)	1 (0.4)	2 (2:2)	2 (60′)	2		1	2	11.46
2	1 (PAM-XG)	2 (0.8)	2 (2:2)	2 (60)	1	2	1	2	21.47
3	1 (PAM-XG)	1 (0.4)	1 (1:3)	1 (0)	1		1		17.02
4	2 (PAM-CMC)	2 (0.8)	3 (3:1)	1 (0)	2	1		2	32.11
5	2 (PAM-CMC)	3 (1.2)	1 (1:3)	2 (60)		1	2		19.42
6	1 (PAM-XG’)	3 (1.2)	2 (2:2)	1 (0)		2	2	1	33.75
7	1 (PAM-XG’)	1 (0.4)	3 (3:1)	2 (60)	2			1	17.45
8	1 (PAM-XG)	3 (1.2)	3 (3:1)	2 (60′)		1		1	27.28
9	1 (PAM-XG’)	2 (0.8)	1 (1:3)	2 (60′)	1	2	2		16.58
K_1_	133.550	45.930	53.020	82.880	55.070	78.810	53.950	69.040	
K_2_	62.990	70.160	66.680	113.660	65.020	71.800	69.750	78.480	
K_3_		80.450	76.840						
k_1_	22.258	15.310	17.673	27.627	18.357	26.270	17.983	23.013	
k_2_	20.997	23.387	22.227	18.943	21.673	23.933	23.250	26.160	
k_3_		26.817	25.613						
Abs. Range	1.262	11.507	7.940	8.683	3.316	2.337	5.267	3.147	

**Table 5:** Results of humidity aging test for individual polymer application.

Runs	Factors				Interactions		PR (N)
	A	B	C	D	C&D	C&D	Averaged
	Polymers	Con. (wt.%)	Aging Time	Humidity Aging Cycle	D from 1 to 2	D from 2 to 3	
**1**	2 (CMC)	1 (0.4)	3 (60′)	2	1	2	3.654
**2**	3 (XG)	3 (1.2)	1 (0)	2	2	1	5.192
**3**	1 (PAM)	1 (0.4)	1 (0)	1	1		11.044
**4**	3 (XG)	2 (0.8)	3 (60′)	1	2		5.463
**5**	2 (CMC)	2 (0.8)	1 (0)	3		2	6.193
**6**	2 (CMC)	3 (1.2)	2 (60)	1	2		7.297
**7**	1 (PAM)	2 (0.8)	2 (60)	2	1	2	6.895
**8**	1 (PAM)	3 (1.2)	3 (60′)	3		1	8.388
**9**	3 (XG)	1 (0.4)	2 (60)	3		1	3.407
**K_1_**	26.327	18.105	22.429	23.804	21.593	16.987	
**K_2_**	17.144	18.551	35.104	15.741	17.952	16.742	
**K_3_**	14.062	20.877		17.988			
**k_1_**	8.776	6.035	7.476	7.935	7.198	5.662	
**k_2_**	5.715	6.184	5.851	5.247	5.984	5.581	
**k_3_**	4.687	6.959	0.000	5.996			
**Abs. Range**	4.088	0.924	1.626	2.688	1.214	0.082	

**Table 6:** Results of temperature aging test for individual polymer application.

Runs	Factors				Interactions		PR (N)
	A	B	C	D	C&D	C&D	Averaged
	Polymers	Con. (wt.%)	Aging Time	Temperature (°C)	D from 1 to 2	D from 2 to 3	
**1**	2 (CMC)	1 (0.4)	3 (60′)	2(45)	1	2	5.611
**2**	3 (XG)	3 (1.2)	1 (0)	2(45)	2	1	8.311
**3**	1 (PAM)	1 (0.4)	1 (0)	1(20)	1		12.068
**4**	3 (XG)	2 (0.8)	3 (60′)	1(20)	2		6.376
**5**	2 (CMC)	2 (0.8)	1 (0)	3(70)		2	11.277
**6**	2 (CMC)	3 (1.2)	2 (60)	1(20)	2	2	8.404
**7**	1 (PAM)	2 (0.8)	2 (60)	2(45)	1		9.982
**8**	1 (PAM)	3 (1.2)	3 (60′)	3(70)		1	8.155
**9**	3 (XG)	1 (0.4)	2 (60)	3(70)		1	5.017
**K_1_**	30.205	22.696	31.656	26.848	27.661	21.483	
**K_2_**	25.292	27.635	43.545	23.904	23.091	25.292	
**K_3_**	19.704	24.870		24.449			
**k_1_**	10.068	7.565	10.552	8.949	9.220	7.161	
**k_2_**	8.431	9.212	7.258	7.968	7.697	8.431	
**k_3_**	6.568	8.290	0.000	8.150			
**Abs. Range**	3.500	1.646	3.295	0.981	1.523	1.270	

**Table 7:** Results of ultraviolet radiation aging test for individual polymer application.

Runs	Factors				Interactions		PR (N)
	A	B	C	D	C&D	C&D	Averaged
	Polymers	Con. (wt.%)	Aging Time	UV Aging Length (Day)	D from 1 to 2	D from 2 to 3	
**1**	2 (CMC)	1 (0.4)	3 (60′)	2(14)	1	2	4.808
**2**	3 (XG)	3 (1.2)	1 (0)	2(14)	2	1	8.347
**3**	1 (PAM)	1 (0.4)	1 (0)	1(7)	1		13.154
**4**	3 (XG)	2 (0.8)	3 (60′)	1(7)	2		6.783
**5**	2 (CMC)	2 (0.8)	1 (0)	3(21)		2	9.374
**6**	2 (CMC)	3 (1.2)	2 (60)	1(7)	2		7.293
**7**	1 (PAM)	2 (0.8)	2 (60)	2(14)	1	2	7.261
**8**	1 (PAM)	3 (1.2)	3 (60′)	3(21)		1	9.017
**9**	3 (XG)	1 (0.4)	2 (60)	3(21)		1	4.717
**K_1_**	29.432	22.679	30.875	27.230	25.223	22.081	
**K_2_**	21.475	23.418	39.879	20.416	21.423	21.443	
**K_3_**	19.847	24.657		23.108			
**k_1_**	9.811	7.560	10.292	9.077	8.408	7.360	
**k_2_**	7.158	7.806	6.647	6.805	7.141	7.148	
**k_3_**	6.616	8.219		7.703			
**Abs. Range**	3.195	0.659	3.645	2.271	1.267	0.213	

**Table 8:** ANOVA analysis of penetration resistance and dynamic viscosity.

Self-Aging	df	Sum Sq	Mean Sq	f Value	p(F)
**Penetration resistance**	1	451.4	451.4	335.6	3.58 × 10^−7^
**Residuals**	7	9.4	1.3		
**Self and humidity aging**	df	Sum Sq	Mean Sq	f value	p(F)
**Penetration resistance**	1	3.8	3.804	0.64	0.45
**Residuals**	7	41.64	5.949		
**Self and temp. aging**	df	Sum Sq	Mean Sq	f value	p(F)
**Penetration resistance**	1	11.13	11.126	2.135	0.187
**Residuals**	7	36.48	5.211		
**Self and UV aging**	df	Sum Sq	Mean Sq	f value	p(F)
**Penetration resistance**	1	5.61	5.614	0.78	0.406
**Residuals**	7	50.36	7.194		

**Table 9:** Composition of red sand powder and red mud leaching solution (wt.%).

Objects	Si	Al	Fe	Ca	Mg	Ti
**Red sand**	21.35	11.21	5.79	35.11	0.98	1.12
**Red mud leaching solution**	0.39	3.57	2.25	8.14	0.69	0.03

## Data Availability

Not applicable.

## References

[1] Xue S., Zhu F., Kong X., Wu C., Huang L., Huang N., Hartley W. (2016). A review of the characterization and revegetation of bauxite residues (Red mud). Environ. Sci. Pollut. Res..

[2] IAI (2012). Background Information on Typical Bauxite Residue.

[3] Howe P.L., Clark M., Reichelt-Brushett A.J., Johnston M. (2011). Toxicity of raw and neutralized bauxite refinery residue liquors to the freshwater cladoceran *Ceriodaphnia dubia* and the marine amphipod *Paracalliope australis*. Environ. Toxicol. Chem..

[4] Huang L., Li Y., Xue S., Zhu F., Wu C., Wang Q. (2016). Salt composition changes in different stacking ages of bauxite residue. Chin. J. Nonferrous Met..

[5] Dauvin J.-C. (2010). Towards an impact assessment of bauxite red mud waste on the knowledge of the structure and functions of bathyal ecosystems: The example of the Cassidaigne canyon (north-western Mediterranean Sea). Mar. Pollut. Bull..

[6] Gelencsér A.S., Kováts N.R., Turóczi B., Rostási A.G., Hoffer A.S., Imre K.L., Nyirő-Kósa I., Csákberényi-Malasics D., Tóth A.D.M., Czitrovszky A.R. (2011). The red mud accident in Ajka (Hungary): Characterization and potential health effects of fugitive dust. Environ. Sci. Technol..

[7] Mayes W., Burke I., Gomes H., Anton Á., Molnár M., Feigl V., Ujaczki É. (2016). Advances in understanding environmental risks of red mud after the Ajka spill, Hungary. J. Sustain. Metall..

[8] Ruyters S., Mertens J., Vassilieva E., Dehandschutter B., Poffijn A., Smolders E. (2011). The Red Mud Accident in Ajka (Hungary): Plant Toxicity and Trace Metal Bioavailability in Red Mud Contaminated Soil. Environ. Sci. Technol..

[9] Kong X., Tian T., Xue S., Hartley W., Huang L., Wu C., Li C. (2017). Development of alkaline electrochemical characteristics demonstrates soil formation in bauxite residue undergoing natural rehabilitation. Land Degrad. Dev..

[10] Zhu F., Cheng Q., Xue S., Li C., Hartley W., Wu C., Tian T. (2017). Influence of natural regeneration on fractal features of residue microaggregates in bauxite residue disposal areas. Land Degrad. Dev..

[11] Power G., Gräfe M., Klauber C. (2011). Bauxite residue issues: I. Current management, disposal and storage practices. Hydrometallurgy.

[12] Klauber C., Gräfe M., Power G. (2011). Bauxite residue issues: II. options for residue utilization. Hydrometallurgy.

[13] Craig K., Nicole H., Renee H., Campbell M. (2016). Proposed Mechanism for the Formation of Dust Horizons on Bauxite Residue Disposal Areas. Light Met..

[14] Ding X., Luo Z., Xu G., Chang P. (2021). Characterization of red sand dust pollution control performance via static and dynamic laboratorial experiments when applying polymer stabilizers. Environ. Sci. Pollut. Res..

[15] Mudgil D., Barak S., Khatkar B.S. (2014). Guar gum: Processing, properties and food applications—A Review. J. Food Sci. Technol..

[16] Santos J., Calero N., Guerrero A., Muñoz J. (2015). Relationship of rheological and microstructural properties with physical stability of potato protein-based emulsions stabilized by guar gum. Food Hydrocoll..

[17] Comba S., Sethi R. (2009). Stabilization of highly concentrated suspensions of iron nanoparticles using shear-thinning gels of xanthan gum. Water Res..

[18] Kolay P.K., Dhakal B. (2019). Geotechnical Properties and Microstructure of Liquid Polymer Amended Fine-Grained Soils. Geotech. Geol. Eng..

[19] Ayeldeen M., Negm A., El-Sawwaf M., Kitazume M. (2017). Enhancing mechanical behaviors of collapsible soil using two biopolymers. J. Rock Mech. Geotech. Eng..

[20] Liu J., Shi B., Jiang H., Huang H., Wang G., Kamai T. (2011). Research on the stabilization treatment of clay slope topsoil by organic polymer soil stabilizer. Eng. Geol..

[21] Zandieh A.R., Yasrobi S.S. (2010). RETRACTED ARTICLE: Study of Factors Affecting the Compressive Strength of Sandy Soil Stabilized with Polymer. Geotech. Geol. Eng..

[22] Genis A., Vulfson L., Ben-Asher J. (2013). Combating wind erosion of sandy soils and crop damage in the coastal deserts: Wind tunnel experiments. Aeolian Res..

[23] Chen R., Ramey D., Weiland E., Lee I., Zhang L. (2016). Experimental Investigation on Biopolymer Strengthening of Mine Tailings. J. Geotech. Geoenviron. Eng..

[24] Ding X., Xu G., Kizil M., Zhou W., Guo X. (2018). Lignosulfonate Treating Bauxite Residue Dust Pollution: Enhancement of Mechanical Properties and Wind Erosion Behavior. Water Air Soil Pollut..

[25] Devrani R., Dubey A.A., Ravi K., Sahoo L. (2021). Applications of bio-cementation and bio-polymerization for aeolian erosion control. J. Arid. Environ..

[26] Ding X., Xu G., Liu W.V., Yang L., Albijanic B. (2019). Effect of polymer stabilizers’ viscosity on red sand structure strength and dust pollution resistance. Powder Technol..

[27] Lemboye K., Almajed A., Alnuaim A., Arab M., Alshibli K. (2011). Improving sand wind erosion resistance using renewable agri-culturally derived biopolymers. Aeolian Res..

[28] Weimin G., Zhiren W., Zhishen W. (2012). Study of mechanism of the w-oh sand fixation. J. Environ. Prot..

[29] Orts W.J., Roa-Espinosa A., Sojka R.E., Glenn G.M., Imam S.H., Erlacher K., Pedersen J.S. (2007). Use of synthetic polymers and bi-opolymers for soil stabilization in agricultural, construction, and military applications. J. Mater. Civ. Eng..

[30] Entry J.A., Sojka R.E. (2000). The Efficacy of Polyacrylamide and Related Compounds to Remove Microorganisms and Nutrients from Animal Wastewater. J. Environ. Qual..

[31] Barakat M. (2011). New trends in removing heavy metals from industrial wastewater. Arab. J. Chem..

[32] Zendehdel M., Barati A., Alikhani H., Hekmat A. (2010). Removal of methylene blue dye from wastewater by adsorption onto semi-inpenetrating polymer network hydrogels composed of acrylamide and acrylic acid copolymer and polyvinyl alcohol. Iran. J. Environ. Health Sci. Eng..

[33] Bjorneberg D.L., Aase J.K. (2000). Multiple polyacrylamide applications for controlling sprinkler irrigation runoff and erosion. Appl. Eng. Agric..

[34] Dou C.-Y., Li F.-H., Wu L. (2012). Soil Erosion as Affected by Polyacrylamide Application Under Simulated Furrow Irrigation with Saline Water. Pedosphere.

[35] He J.-J., Cai Q.-G., Tang Z.-J. (2007). Wind tunnel experimental study on the effect of PAM on soil wind erosion control. Environ. Monit. Assess..

[36] Bergmann D., Furth G., Mayer C. (2008). Binding of bivalent cations by xanthan in aqueous solution. Int. J. Biol. Macromol..

[37] Debnath B., Das B. (2020). Influence of hydrophobic and electrostatic interactions on counterion-dissociation in sodium carbox-ymethylcellulose—Polyethylene glycol (PEG) solutions. J. Mol. Liq..

[38] Koneru A., Dharmalingam K., Anandalakshmi R. (2020). Cellulose based nanocomposite hydrogel films consisting of sodium carboxymethylcellulose–grapefruit seed extract nanoparticles for potential wound healing applications. Int. J. Biol. Macromol..

[39] Tang J., Fan C., Lin Q., Zhou X. (2014). Smooth, stable and optically transparent microcapsules prepared by one-step method using sodium carboxymethyl cellulose as protective colloid. Colloids Surf. A Physicochem. Eng. Asp..

[40] Shakun M., Heinze T., Radke W. (2013). Determination of the DS distribution of non-degraded sodium carboxymethyl cellulose by gradient chromatography. Carbohydr. Polym..

[41] ASTM (2011). D2487-17, Standard Practice for Classification of Soils for Engineering Purposes (Unified Soil Classification System).

[42] Australian Standard (2005). 1289.2.1.1, Methods of Testing Soils for Engineering Purposes-Soil Moisture Content Tests-Determination of the Moisture Content of a Soil-Oven Drying Method (Standard Method).

[43] Australian Standard (2009). 1289.3.6.1, Standard Method of Analysis by Sieving in Methods of Testing Soils for Engineering Purpos-es—Soil Classification Tests—Determination of the Particle Size Distribution of a Soil.

[44] Australian Standard (1998). AS1289.6.2.2, Soil Strength and Consolidation Tests—Determination of the Shear Strength of a Soil—Direct Shear Test Using a Shear Box: Methods of Testing Soils for Engineering Purposes.

[45] ASTM (2016). ASTM G154-16, Standard Practice for Operating Fluorescent Ultraviolet (UV) Lamp Apparatus for Exposure of Non-Metallic Materials.

[46] Abdullah N., Chin N.L., Yusof Y.A., Talib R.A. (2017). Modeling the rheological behavior of thermosonic extracted guava, pomelo, and soursop juice concentrates at different concentration and temperature using a new combination model. J. Food Process. Preserv..

[47] Wu Q. (2013). The dividing concentrations of polymer solutions in the Lytropic condensation of macromolecules. Chin. Polym. Bull..

